# Efficacy of neoadjuvant therapy and lymph node dissection in advanced gallbladder cancer without distant metastases: a SEER database analysis

**DOI:** 10.3389/fonc.2024.1511583

**Published:** 2024-11-25

**Authors:** Jun Dong, Zhengqiu Zhu

**Affiliations:** ^1^ Xuzhou Medical University, Xuzhou, Jiangsu, China; ^2^ Department of Medical Oncology, Affiliated Hospital of Xuzhou Medical University, Xuzhou, Jiangsu, China

**Keywords:** neoadjuvant therapy, radical resection, stage III/IV gallbladder cancer, lymph node dissection, survival analysis, SEER database

## Abstract

**Purpose:**

To investigate the effectiveness of neoadjuvant therapy and lymph node dissection(LND) on overall survival (OS) in patients with stage III/IV gallbladder cancer without distant metastases.

**Methods:**

Data from 101 patients who received neoadjuvant therapy followed by surgery combined with adjuvant chemotherapy, and 1412 patients who received direct surgical treatment followed by adjuvant chemotherapy, were collected from the SEER database from 2004 to 2020. Patients were divided into group A (neoadjuvant therapy) and group B (direct surgery) based on the treatment modality. A total of 202 cases were obtained after propensity score matching, with 101 cases in each group (A and B). Cox unifactorial and multifactorial analyses were performed to identify independent risk factors for patients with advanced cholecystic carcinoma, and the Kaplan-Meier method was used to analyze overall survival (OS). The Cox proportional hazards model was used to investigate the effect of different subgroups on OS in both patient groups. Further survival analyses were conducted to determine whether lymph node dissection(LND) was beneficial for patients receiving neoadjuvant therapy for gallbladder cancer.

**Results:**

Cox univariate analysis showed that marital status, AJCC stage, number of LND, tumor size, and treatment modality were associated with OS (P<0.05). Cox multifactorial regression analysis indicated that AJCC stage, LND, tumor size, and treatment modality were independent risk factors for OS in patients with non-metastatic advanced gallbladder cancer (P<0.05). Survival curves demonstrated that the OS in group A was longer than in group B (median OS: 30 months vs. 14 months, P<0.001). Subgroup analysis indicated that neoadjuvant therapy had a consistent effect on the OS of patients with advanced gallbladder cancer, improving both survival time and outcomes. Survival curves indicated that lymph node dissection was not significant in group A patients (p>0.05) but was significant in group B (p<0.05).

**Conclusion:**

Neoadjuvant therapy can improve the OS of patients with non-metastatic stage III/IV gallbladder cancer and is an independent risk factor affecting prognosis; however, the significance of lymph node dissection in these patients still needs further study.

## 1 Introduction

Gallbladder cancer is an aggressive malignant tumor, and due to its unique anatomical structure, subtle symptoms, and susceptibility to lymph node metastasis, most patients are already in stage III/IV at the time of consultation (1). Radical surgical resection is considered the only possible cure for gallbladder cancer (2, 3). However, for patients with stage III gallbladder cancer, radical resection is often not feasible due to the tumor’s invasive location, the patient’s systemic condition, and the inability to achieve R0 resection (4). No consensus exists on the further treatment of such patients. The role of radical surgical resection for patients with stage IV gallbladder cancer without distant metastases remains controversial (5). Neoadjuvant therapy aims to reduce tumor volume and achieve tumor downstaging through preoperative systemic therapy, thereby improving the success rate of R0 resection (6, 7). Neoadjuvant therapy is currently widely used in breast (8), ovarian (9), gastric (10), and colorectal cancers (11). Although neoadjuvant therapies have been shown to enhance overall survival in some malignancies, no completed large phase III clinical trial has conclusively demonstrated their therapeutic benefits in advanced gallbladder cancer. In this study, a propensity score matching (PSM) analysis was conducted using the SEER database to investigate the effectiveness of neoadjuvant therapy on the OS of patients with stage III/IV gallbladder cancer without distant metastases.

## 2 Methods

### 2.1 Patient selection

**Figure 1:**
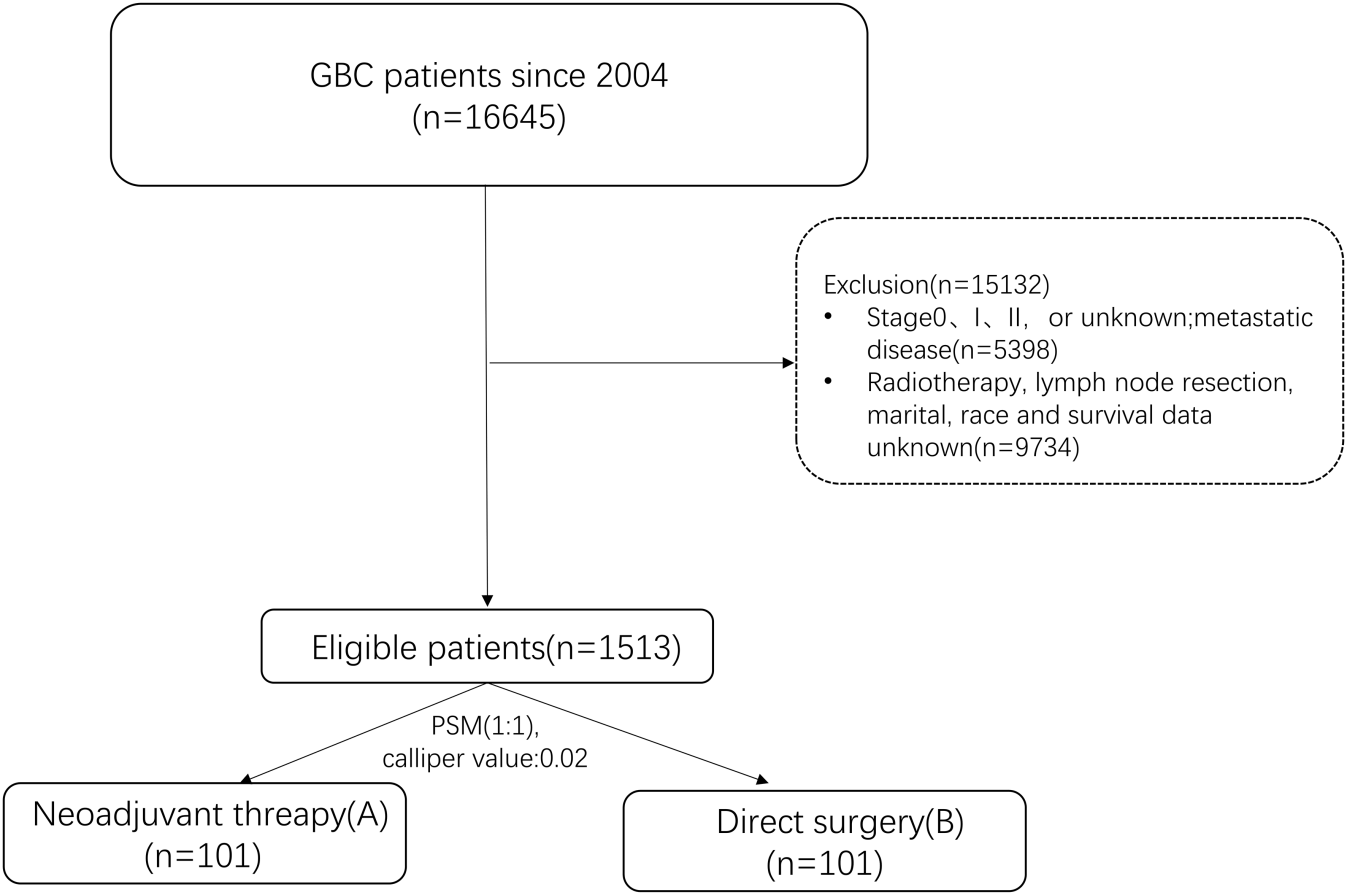
Relationship Flowchart for the screening of research subjects.

Data were from SEER*Stat 8.4.3 software and included patient demographics, clinicopathological, and treatment information. The inclusion criteria: (1) Primary tumor located in the gallbladder (anatomical code: C23.9); (2) Year of diagnosis from 2004 to 2020; (3) International Classification of Diseases for Oncology ICDO-3 code [adenocarcinoma (8140–8389), other]; (4) Patients with non-distant metastases (Stage III/IV), classified as cT1-cT4, cN0-2, and cM0, were re-staged according to the 8th edition of the AJCC staging system; (5) Use of ‘RX Summ - Systemic/Sur Seq’, “RX Summ - Surg Prim Site (1998+)”, ‘Chemotherapy recode,’ and ‘RX Summ–Surg/Rad Seq’ fields to screen patients who received neoadjuvant therapy followed by surgery combined with chemotherapy or direct surgical treatment followed by adjuvant chemotherapy; (6) All patients underwent radical surgical resection. The exclusion criteria: (1) Distant metastases; (2) Unknown surgical information or no surgical treatment; (3) Incomplete prognostic or clinical information.1513 patients were finally screened for the study (**Figure 1**). Patients were divided into group A (neoadjuvant therapy) and group B (direct surgery) based on the treatment modality.

### 2.2 Data collection

The data extracted from the SEER database comprised variables such as gender, age at diagnosis, race, marital, histology, grade, the clinical T-stage, the clinical N-stage, AJCC, surgical treatment, radiotherapy, chemotherapy, survival time, and survival status.

### 2.3 Statistical analysis

Relevant data were analyzed and visualized utilizing SPSS 26 and R 4.3.3. Categorical variables were compared using the chi-square test or Fisher’s exact test. To minimize bias between group A and group B, propensity score matching (1:1) was performed with a caliper value of 0.02. Independent risk factors for OS were identified through Cox univariate and multivariate analyses. Survival curves were generated utilizing the ‘survival’ and ‘survminer’ packages, and the log-rank test was utilized to assess the predictive value of efficacy between groups. Forest plots for subgroups were created using the ‘forestploter’ package.

## 3 Results

### 3.1 Comparison of baseline information between the two groups before and after PSM respectively

Statistically meaningful differences were found between the two groups before PSM, with 101 people in A and 1412 people in B. Significant differences were found in grade classification, T stage, AJCC stage, lymph node clearance, and tumor size. After PSM, the baseline data of the two groups were balanced, resulting in 101 matched pairs in group B for group A; see **Table 1** for details.

**Table 1:** Baseline data for patients with stage III/IV gallbladder cancer without distant metastasis.

PSM Before						PSM After				
Item	Overall	groupA	groupB	X^2^	P	Overall	groupA	groupB	X^2^	P
	(N=1513)	(N=101)	(N=1412)			(N=202)	(N=101)	(N=101)		
Age				0.010	0.917				0.554	0.456
<60	450(29.7%)	31 (30.7%)	419 (29.7%)			68 (33.7%)	31 (30.7%)	37 (36.6%)		
≥60	1063 (70.3%)	70 (69.3%)	993 (70.3%)			134 (66.3%)	70 (69.3%)	64 (63.4%)		
Gender				0.660	0.416				0.199	0.655
Female	1094 (72.3%)	69 (68.3%)	1025 (72.6%)			134 (66.3%)	69 (68.3%)	65 (64.4%)		
Male	419 (27.7%)	32 (31.7%)	387 (27.4%)			68 (33.7%)	32 (31.7%)	36 (35.6%)		
Race				0.776	0.678				2.533	0.281
Black	198 (13.1%)	11 (10.9%)	187 (13.2%)			30 (14.9%)	11 (10.9%)	19 (18.8%)		
Others	179 (11.8%)	14 (13.9%)	165 (11.7%)			26 (12.9%)	14 (13.9%)	12 (11.9%)		
White	1136 (75.1%)	76 (75.2%)	1060 (75.1%)			146 (72.3%)	76 (75.2%)	70 (69.3%)		
Marital				1.657	0.198				2.777	0.249
Married	1222 (80.8%)	87 (86.1%)	1135 (80.4%)			158 (78.2%)	87 (86.1%)	71 (70.3%)		
Single	291 (19.2%)	14 (13.9%)	277 (19.6%)			44 (21.8%)	14 (13.9%)	30 (29.7%)		
Histologic				1.978	0.159				0.032	0.857
Others	220 (14.5%)	20 (19.8%)	200 (14.2%)			38 (18.8%)	20 (19.8%)	18 (17.8%)		
Adeno	1293 (85.5%)	81 (80.2%)	1212 (85.8%)			164 (81.2%)	81 (80.2%)	83 (82.2%)		
Grade				13.717	0.002				0.298	0.585
Grade I-II	726 (48.0%)	30 (29.7%)	696 (49.3%)			77 (38.1%)	30 (29.7%)	47 (46.5%)		
Grade III-IV	787 (52.0%)	71 (70.3%)	716 (50.7%)			125 (61.9%)	71 (70.3%)	54 (53.5%)		
Clinical T stage				12.749	0.003				0.031	0.859
cT1-2	541 (35.8%)	19 (18.8%)	522 (37.0%)			40 (19.8%)	19 (18.8%)	21 (20.8%)		
cT3-4	972 (64.2%)	82 (81.2%)	890 (63.0%)			162 (80.2%)	82 (81.2%)	80 (79.2%)		
Clinical N stage				1.178	0.554				1.277	0.527
N0	685 (45.3%)	42 (41.6%)	643 (45.5%)			92 (45.5%)	42 (41.6%)	50 (49.5%)		
N1	752 (49.7%)	52 (51.5%)	700 (49.6%)			97 (48.0%)	52 (51.5%)	45 (44.6%)		
N2	76 (5.0%)	7 (6.9%)	69 (4.9%)			13 (6.4%)	7 (6.9%)	6 (5.9%)		
AJCC				4.803	0.028				0.000	1.000
III	673 (44.5%)	56 (55.4%)	617 (43.7%)			112 (55.4%)	56 (55.4%)	56 (55.4%)		
IV	840 (55.5%)	45 (44.6%)	795 (56.3%)			90 (44.6%)	45 (44.6%)	45 (44.6%)		
LND				13.648	0.001				1.408	0.494
0	530 (35.0%)	19 (18.8%)	511 (36.2%)			41 (20.3%)	19 (18.8%)	22 (21.8%)		
1-3	565 (37.3%)	43 (42.6%)	522 (37.0%)			91 (45.0%)	43 (42.6%)	48 (47.5%)		
≥4	418 (27.6%)	39 (38.6%)	379 (26.8%)			70 (34.7%)	39 (38.6%)	31 (30.7%)		
Radiation				1.243	0.264				0.024	0.875
No	1155 (76.3%)	72 (71.3%)	1083 (76.7%)			146 (72.3%)	72 (71.3%)	74 (73.3%)		
Yes	358 (23.7%)	29 (28.7%)	329 (23.3%)			56 (27.7%)	29 (28.7%)	27 (26.7%)		
Tumor size				6.369	0.041				0.439	0.802
<5cm	846 (55.9%)	45 (44.6%)	801 (56.7%)			88 (43.6%)	45 (44.6%)	43 (42.6%)		
≥5cm	294 (19.4%)	22 (21.8%)	272 (19.3%)			48 (23.8%)	22 (21.8%)	26 (25.7%)		
Unknown	373 (24.7%)	34 (33.7%)	339 (24.0%)			66 (32.7%)	34 (33.7%)	32 (31.7%)		

### 3.2 Unifactorial and multifactorial analyses affecting OS in patients with non-metastatic gallbladder cancer

On Cox univariate analysis, marital status, AJCC stage, LND, tumor size, and treatment modality were meaningfully associated with OS. On Cox multivariate analysis, AJCC stage, LND, tumor size, and treatment modality were independent risk factors. Stage IV people had a worse prognosis than stage III patients (HR: 1.758, 95% CI: 1.311-2.358, P < 0.001). Patients with 1-3 lymph nodes resected and those with ≥ 4 lymph nodes resected had a better prognosis (HR: 0.494, 95% CI: 0.331-0.739, P < 0.001) than patients with no lymph nodes removed (HR: 0.453, 95% CI: 0.295-0.695, P < 0.001). Additionally, patients with tumor size (<5cm) had a better prognosis. Compared with patients receiving neoadjuvant therapy, those undergoing direct surgery had a worse prognosis (HR: 2.322, 95% CI: 1.681-3.209, P < 0.001). For details, see **Table 2**.

**Table 2:** COX regression analysis of OS in patients with gallbladder cancer.

Item	Univariate analysis		Multivariate analysis	
	HR(95%CI)	P	HR(95%CI)	P
Age				
<60	Reference			
≥60	0.774(0.572-1.048)	0.166		
Gender				
Female	Reference			
Male	0.992(0.730-1.347)	0.968		
Race				
Black	Reference			
Others	0.991(0.564-1.741)	0.979		
White	1.067(0.711-1.601)	0.791		
Marital				
Married	Reference		Reference	
Single	1.100(1.066-1.579)	0.043	1.056(0.601-1.414)	0.757
Histologic				
Others	Reference			
Adeno	0.876(0.603-1.272)	0.562		
Grade				
Grade I-II	Reference			
Grade III-IV	1.100(0.817-1.482)	0.596		
Clinical T stage				
T1-2	Reference			
T3-4	1.243(0.856-1.805)	0.336		
Clinical N stage				
N0	Reference			
N1	0.979(0.726-1.319)	0.909		
N2	0.821(0.441-1.527)	0.602		
AJCC				
III	Reference		Reference	
IV	1.758(1.311-2.358)	<0.001	1.91(1.408-2.593)	<0.001
LND				
0	Reference		Reference	
1-3	0.529(0.367-0.763)	0.004	0.494(0.331-0.739)	0.004
≥4	0.494(0.334-0.730)	0.002	0.453(0.295-0.695)	0.002
Radiation				
No	Reference			
Yes	0.938(0.681-1.293)	0.745		
Tumor size				
<5cm	Reference		Reference	
≥5cm	1.911(1.327-2.751)	0.003	2.042(1.402-2.975)	0.002
Unknown	1.304(0.927-1.835)	0.199	1.195(0.835-1.710)	0.412
Treatment				
groupA	Reference		Reference	
groupB	1.909(1.417-2.571)	0.003	2.322(1.680-3.209)	<0.001

### 3.3 Survival curves in patients

Neoadjuvant therapy has been found to have a remarkable impact on people’s OS. The survival curves indicated that the OS in A was longer than in B (median OS: 30 months vs. 14 months, P<0.001). Further analysis was conducted to assess the effect of neoadjuvant therapy on OS across various patient subgroups, based on independent risk factors identified from a multifactorial Cox proportional hazards model. The results demonstrated that neoadjuvant therapy resulted in a longer median survival time compared to group B across different stages: stage III (median OS: 31 months vs. 14 months, P<0.001) and stage IV (median OS: 25 months vs. 10 months, P<0.001). For lymph node dissection (LND), the median OS was: LND=0 (16 months vs. 7 months, P<0.001), LND=1-3 (30 months vs. 15 months, P<0.001), and LND≥4 (21 months vs. 25 months, P=0.21). Regarding tumor size, <5 cm (38 months vs. 17 months, P<0.001), ≥5 cm (22 months vs. 10 months, P<0.001), and unknown tumor size (19 months vs. 21 months, P=0.28).

**Figure 2:**
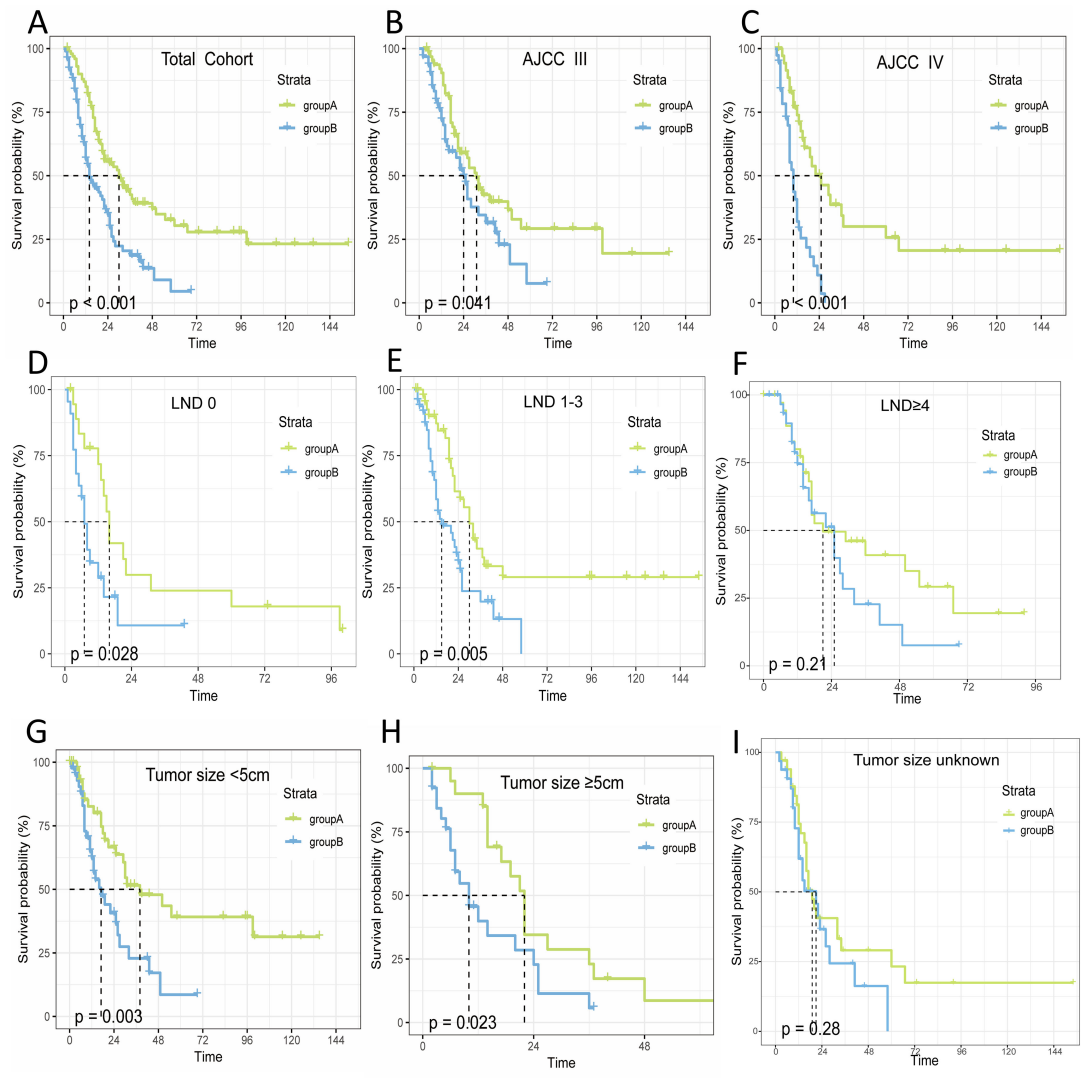
Relationship between OS and treatment modalities in patients with non-metastatic advanced gallbladder cancer. **(A)** Total; **(B)** StageIII; **(C)** StageIV; **(D)** LND=0; **(E)** LND=1-3; **(F)** LND≥4; **(G)** Tumor size<5cm; **(H)** Tumor size≥5cm; **(I)** Tumor size unknown.

Overall, the OS in group A was longer than in group B across different stages (stage III/IV), lymph node dissection (LND=0/1-3), and tumor sizes (<5 cm/≥5 cm). No significant difference in OS was observed for LND≥4 and unknown tumor size (P>0.05), as illustrated in **Figure 2**.

### 3.4 Subgroup analysis of treatment modalities in patient OS

**Figure 3:**
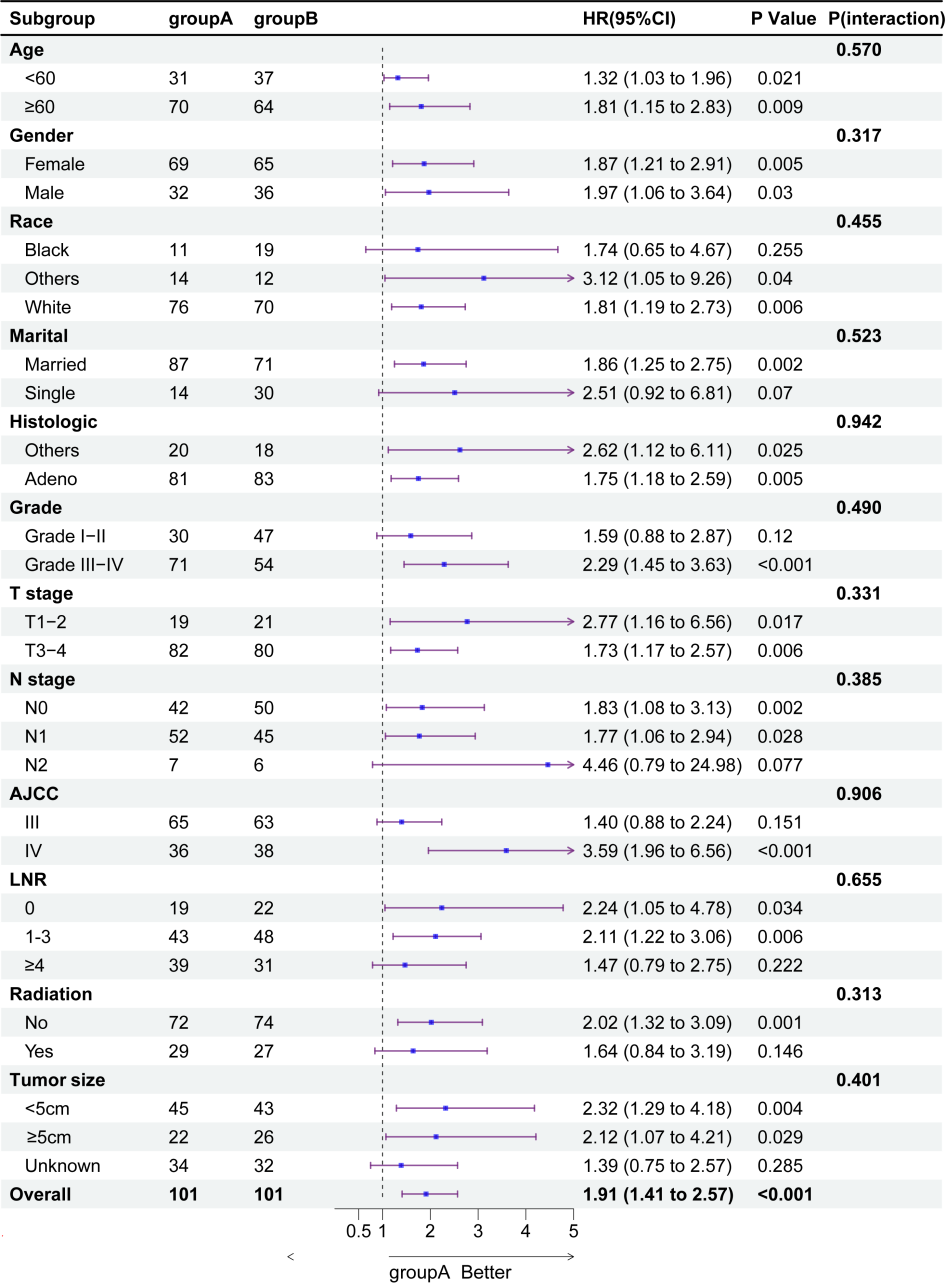
Subgroup analysis of overall survival in patients with gallbladder cancer.

To illustrate the interaction between other risk factors and treatment modalities, we performed 12 subgroup analyses on the total cohort. **Figure 2** shows that neoadjuvant therapy was significantly associated with improved OS prognosis. The forest plot for the subgroup analyses (**Figure 3**) showed consistent proportional effects in the OS analyses, with no heterogeneity found in all 12 prespecified subgroups (P > 0.05 for all interactions).

### 3.5 Survival analysis of lymph node dissection regarding N0 and N1/N2 patients

**Figure 4:**
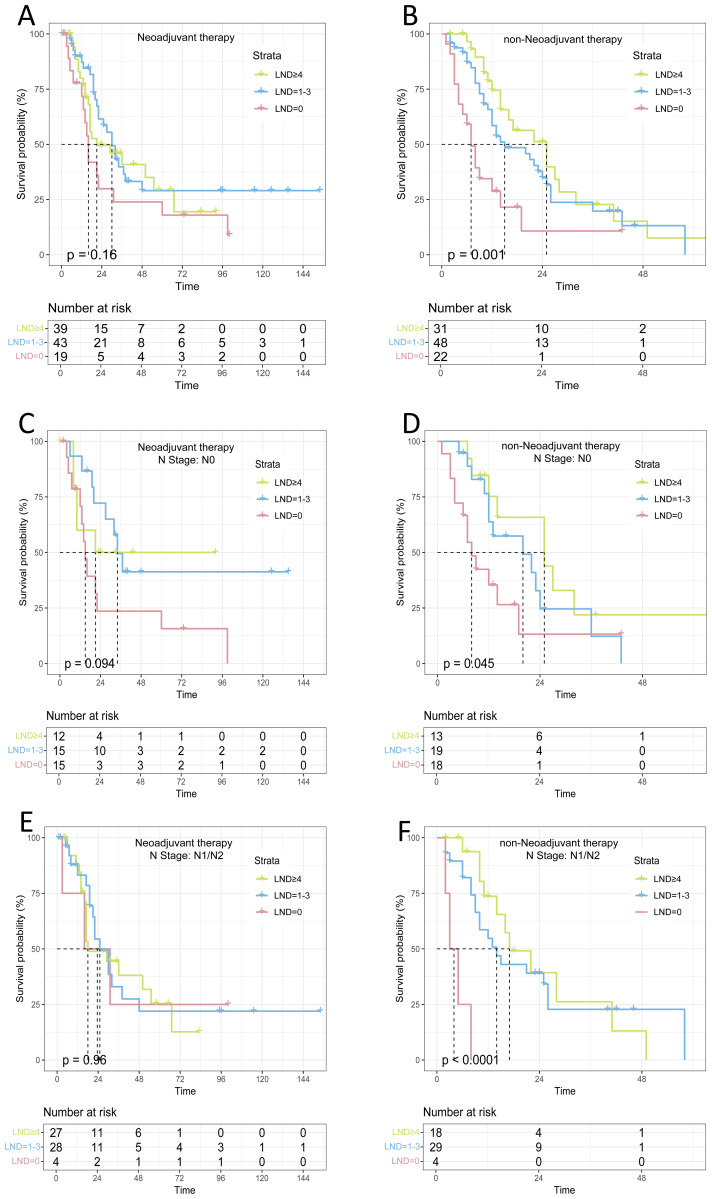
Survival analysis of lymph node dissection with different treatment modalities and lymph node dissection **(A)** Neoadjuvant therapy; **(B)** non-Neoadjuvant therapy; **(C)** Neoadjuvant therapy(N0); **(D)** non-Neoadjuvant therapy(N0); **(E)** Neoadjuvant therapy(N1/N2); **(F)** non-Neoadjuvant therapy(N1/N2);.

Lymph node dissection (LND) is an independent risk factor for patients with advanced non-metastatic gallbladder cancer. However, our COX regression analyses of different treatment subgroups revealed that LND was an independent prognostic factor for patients in the direct surgery group (P<0.05), but not for those in the neoadjuvant group (P>0.05), **Supplementary Tables 1, 2**. Kaplan-Meier survival curves indicated that lymph node dissection (LND) provided a survival benefit in the direct surgery group (P=0.001), while no significant survival benefit was observed in the neoadjuvant therapy group (P>0.05). We found that in N0 patients receiving neoadjuvant therapy, the median OS for the LND=0, LND=1-3, and LND≥4 groups was 15 months, 34 months, and 21 months, respectively, with no statistically significant difference (P>0.05). In N1/N2 patients receiving neoadjuvant therapy, the median OS for the LND=0, LND=1-3, and LND≥4 groups was 18 months, 25 months, and 23.5 months, respectively, with no statistically significant difference (P>0.05). However, among patients without neoadjuvant therapy, the median OS for the three groups of N0 patients was 8, 20, and 25 months, respectively, with a statistically significant difference (P=0.045). The median OS for the three groups of N1 patients was 4, 14, and 17 months, with a remarkably meaningful difference (P<0.001), as shown in **Figure 4**.

## 4 Discussion

Radical surgical resection is an important therapy for advanced gallbladder cancer; however, only 10% of patients have the opportunity for surgical treatment (4). Additionally, direct surgical treatment of advanced patients without distant metastases has a low R0 resection rate and is prone to high postoperative recurrence. Gong Wei’s team conducted a study of gallbladder cancer in China, involving 6159 cases. The study showed that 34.26% of patients had lost the opportunity for surgery at the time of their initial consultation. Among those who underwent surgical treatment, 58.89% achieved R0 resection, leaving a high percentage of patients who did not achieve radical resection (12). Therefore, direct surgical resection is not an appropriate option for people.

With the increasing adoption of neoadjuvant therapy across various malignancies, it is also becoming a viable treatment option for people with advanced gallbladder cancer. A single-center retrospective research at the University of Montreal, Canada, showed that only 28.6% of patients with T3 GBC achieved R0 resection (13). An international multicenter study (14) initiated by the Anderson Cancer Centre showed that T3 to T4 stage was an independent factor in the recurrence of GBC, with a recurrence rate of up to 40% of patients at 1 year after surgery. Creasy et al. (15) found that 45% of patients could undergo radical resection following neoadjuvant therapy. The OS was different between the R0 resection group and others, with the former achieving 51 months compared to 11 months in the latter. Our study yielded comparable results, showing that the OS for patients in A was longer than that for patients in B (median OS: 25 months vs. 14 months, P<0.001). Multivariate analysis further identified neoadjuvant treatment as an independent prognostic factor in advanced gallbladder cancer (P<0.001). This indicates that neoadjuvant therapy enhances OS for people with gallbladder cancer without distant metastases. However, while a similar benefit was observed for OS in patients with locally advanced gallbladder cancer, the results did not reach statistical meaning (P>0.05) (16). Fareed et al. (17) conducted retrospective research on the OS of people with non-metastatic gallbladder cancer who received neoadjuvant radiotherapy and found no benefit in subgroup analysis. Consequently, the efficacy of neoadjuvant therapy in patients with advanced gallbladder cancer remains a topic of debate. The GAIN trial will confirm the good points of neoadjuvant therapy in gallbladder cancer. The results, expected to be published this year, will provide a higher level of evidence-based support for the use of neoadjuvant therapy in advanced gallbladder cancer (18).

Additionally, this study identified lymph node dissection as an independent prognostic factor for patients who did not receive neoadjuvant therapy, as determined by Cox regression analysis. Maegawa et al. (19) conducted a prognostic analysis of gallbladder cancer and reported that the OS rate of patients who did not undergo lymphatic clearance was lower than that of patients with positive lymph node metastases after clearance (HR=1.11, 95% CI 1.01-1.22). Tumor size and AJCC stage were also independent risk factors for patients, consistent with previous studies (20, 21). However, there are fewer studies on whether LND prolongs OS in patients who have undergone surgical resection after neoadjuvant therapy. In the present study, we analyzed the OS of patients who received neoadjuvant therapy and found no meaningful difference between patients with and without lymph node metastasis (P>0.05). However, there was a meaningful difference in survival among patients without neoadjuvant therapy based on lymph node metastasis status (P<0.05). Ito et al. (22), by retrospectively analyzing the data of 122 patients with gallbladder cancer, concluded that intraoperative clearance of ≥6 lymph nodes significantly improved patients’ prognosis, a conclusion shared by other scholars (23). Widmann et al. (24), through a literature review, found that LND improved the OS of patients with gallbladder cancer and that clearing at least 6 lymph nodes significantly improved patients’ prognosis. However, these studies were conducted in patients with gallbladder cancer who did not receive neoadjuvant therapy, and no large studies have been conducted in patients who received neoadjuvant therapy. We hypothesize that the differences in lymph node dissection outcomes between different treatment modalities may be due to the number of LND in this study being classified as ≥4 based on SEER database data, which may differ from previous classifications. Secondly, the lymph node status of a patient may change after receiving neoadjuvant therapy. Additionally, the small sample size of this study may have introduced a larger bias. Therefore, future multicenter, large-scale prospective researches are needed to investigate the significance of LND and the numbers of LND in the prognosis of patients undergoing radical resection after neoadjuvant therapy. There is an urgent need to explore prognostic biomarkers for patients undergoing neoadjuvant therapy. Currently, circulating tumor DNA and solid tumor microscopic residual disease (MRD) has shown significant value in lung, breast, and colon cancers (25). Other biomarkers, such as TTK (26), IL-22 (27), and the oncogenic Neuregulin 1 gene (NRG1) (28), have also demonstrated better predictive value in various solid tumors (29, 30). However, more research is required to determine their effectiveness in patients with advanced gallbladder cancer receiving neoadjuvant therapy.

Currently, there is no preferred neoadjuvant treatment option for gallbladder cancer (GBC). The gemcitabine + cisplatin (GC) regimen has been recommended as the first-line standard chemotherapy for biliary tract cancers (BTC) since the results of the ABC-02 trial (31). The 2023 National Comprehensive Cancer Network (NCCN) guidelines (32) recommend a gemcitabine-based combination chemotherapy regimen for gallbladder cancer. The combination regimens include GC, 5-fluorouracil (5-FU) + oxaliplatin + calcium folinate (FOLFOX), capecitabine + oxaliplatin, gemcitabine + capecitabine, durvalumab + gemcitabine + cisplatin, and gemcitabine + cisplatin + albumin paclitaxel. Gong Wei’s team is conducting a phase II clinical study on the combination of gemcitabine and albumin-paclitaxel for the treatment of progressive gallbladder cancer, with preliminary results showing an ORR of 48% (12). Radiotherapy in the neoadjuvant treatment of gallbladder cancer is often combined with chemotherapy. Engineer et al. (33) reported that people with T3 and T4 gallbladder cancer had the opportunity for R0 resection after gemcitabine monotherapy combined with high-dose radiotherapy, achieving a significant survival benefit. Fareed et al. (17) retrospectively analyzed patients with non-metastatic gallbladder and bile duct cancers who had undergone radical surgery at a single center and did not observe a significant survival benefit from neoadjuvant radiotherapy in gallbladder cancer patients. The POLCAGB study (34) is an ongoing phase III randomized clinical trial comparing neoadjuvant chemotherapy to neoadjuvant radiotherapy for the treatment of T3 and T4 gallbladder cancers. This study is expected to provide high-level evidence-based guidance for the use of neoadjuvant radiotherapy in gallbladder cancer and inform clinical practice.

Numerous limitations were identified in this study. Firstly, data were unavailable on patients who underwent neoadjuvant therapy and then experienced disease progression or were otherwise unable to undergo surgery, and data on postoperative R0 resection rates and changes in lymph node status after neoadjuvant therapy were difficult to obtain. Secondly, the stability of the conclusions of this study was affected by the small sample size. Thirdly, the study data were derived from the SEER database, which did not allow access to all relevant variables, resulting in selection bias. Fourthly, as a retrospective study, data collection and analyses were subject to bias. Finally, information on specific protocols for neoadjuvant therapy was lacking, which is crucial for clinical practice.

In conclusion, the results of this study indicated that the neoadjuvant therapy group exhibited a better prognosis than the direct surgery group. Future research should investigate in greater depth the impact of different neoadjuvant treatment regimens on the survival of patients with stage III/IV gallbladder cancer who have not developed distant metastases, to determine the treatment regimen with the optimal outcome. Secondly, no survival benefit of lymph node dissection was found in patients receiving neoadjuvant therapy, necessitating large-scale studies to confirm the impact of lymph node dissection on survival in neoadjuvant-treated patients, as well as multicenter trials to assess the generalizability of the results to different populations, and long-term follow-up studies to assess the durability of the survival benefit. Finally, the cost-effectiveness of neoadjuvant therapy and lymph node dissection needs further evaluation.

## 5 Conclusion

Neoadjuvant therapy can improve the OS of patients with non-metastatic stage III/IV gallbladder cancer and is an independent risk factor; however, the significance of lymph node dissection in these patients still needs further study.

## Data Availability

The original contributions presented in the study are included in the article/**Supplementary Material**. Further inquiries can be directed to the corresponding author.
