# Supplementary material for: Efficacy of neoadjuvant therapy and lymph node dissection in advanced gallbladder cancer without distant metastases: a SEER database analysis
Source: Front Oncol. 2024 Nov 25;14:1511583. doi: 10.3389/fonc.2024.1511583 (PMC11625672; doi:10.3389/fonc.2024.1511583)
Supplement: Supplementary file 1 [file Table1.docx]

Supplementary Material

# Supplementary Tables

Supplementary Table 1 COX regression analysis in patients not receiving neoadjuvant therapy

| Variables | Univariate analysis |  | Multivariate analysis |  |
| --- | --- | --- | --- | --- |
|  | HR (95%CI) | P | HR (95%CI) | P |
| Age |  |  |  |  |
| <60 | Reference |  |  |  |
| ≥60 | 0.72 (0.43-1.18) | 0.186 |  |  |
| Gender |  |  |  |  |
| Female | Reference |  |  |  |
| Male | 0.79 (0.49-1.28) | 0.339 |  |  |
| Race |  |  |  |  |
| Black | Reference |  |  |  |
| Others | 0.39 (0.15-1.01) | 0.052 |  |  |
| White | 0.56 (0.28-1.12) | 0.101 |  |  |
| Marital |  |  |  |  |
| Married | Reference |  | Reference |  |
| Single | 2.18 (1.19-3.96) | 0.011 | 1.40 (0.73-2.69) | 0.313 |
| Histologic |  |  |  |  |
| Others | Reference |  |  |  |
| Adeno | 0.55 (0.29-1.02) | 0.058 |  |  |
| Grade |  |  |  |  |
| Grade I-II | Reference |  |  |  |
| Grade III-IV | 1.45 (0.90-2.36) | 0.131 |  |  |
| Clinical T stage |  |  |  |  |
| T1-2 | Reference |  |  |  |
| T3-4 | 1.14 (0.65-2.02) | 0.65 |  |  |
| Clinical N stage |  |  |  |  |
| N0 | Reference |  |  |  |
| N1 | 0.97 (0.59-1.57) | 0.888 |  |  |
| N2 | 1.23 (0.43-3.49) | 0.695 |  |  |
| AJCC |  |  |  |  |
| III | Reference |  | Reference |  |
| IV | 3.38 (2.02-5.66) | <0.001 | 3.21 (1.81-5.68) | <0.001 |
| LND |  |  |  |  |
| 0 | Reference |  | Reference |  |
| 1-3 | 0.44 (0.24-0.79) | 0.006 | 0.46 (0.24-0.87) | 0.016 |
| ≥4 | 0.33 (0.17-0.64) | 0.001 | 0.28 (0.14-0.58) | <0.001 |
| Radiation |  |  |  |  |
| No | Reference |  |  |  |
| Yes | 0.81 (0.47 ~ 1.39) | 0.447 |  |  |
| Tumor size |  |  |  |  |
| <5cm | Reference |  | Reference |  |
| ≥5cm | 1.88 (1.05 ~ 3.38) | 0.033 | 2.68 (1.43-5.03) | 0.002 |
| Unknown | 0.98 (0.56 ~ 1.73) | 0.947 | 1.00 (0.55-1.83) | 0.988 |

Supplementary Table 2 COX regression analysis in patients receiving neoadjuvant therapy

| Variables | Univariate analysis |  | Multivariate analysis |  |
| --- | --- | --- | --- | --- |
|  | HR (95%CI) | P | HR (95%CI) | P |
| Age |  |  |  |  |
| <60 | Reference |  |  |  |
| ≥60 | 0.81 (0.48-1.38) | 0.446 |  |  |
| Gender |  |  |  |  |
| Female | Reference |  |  |  |
| Male | 0.77 (0.43-1.38) | 0.383 |  |  |
| Race |  |  |  |  |
| Black | Reference |  |  |  |
| Others | 1.36 (0.52-3.55) | 0.526 |  |  |
| White | 1.29 (0.63-2.66) | 0.488 |  |  |
| Marital |  |  |  |  |
| Married | Reference |  |  |  |
| Single | 0.97 (0.54-1.75) | 0.932 |  |  |
| Histologic | |  |  |  |
| Others | Reference |  |  |  |
| Adeno | 1.11 (0.58-2.14) | 0.75 |  |  |
| Grade |  |  |  |  |
| Grade I-II | Reference |  |  |  |
| GradeIIIIV | 1.02 (0.59-1.74) | 0.948 |  |  |
| Clinical T stage | |  |  |  |
| T1-2 | Reference |  |  |  |
| T3-4 | 1.66 (0.79-3.49) | 0.182 |  |  |
| Clinical N stage | |  |  |  |
| N0 | Reference |  |  |  |
| N1 | 1.07 (0.63-1.81) | 0.8 |  |  |
| N2 | 0.76 (0.26-2.19) | 0.614 |  |  |
| AJCC |  |  |  |  |
| III | Reference |  |  |  |
| IV | 1.17 (0.70-1.97) | 0.552 |  |  |
| LND |  |  |  |  |
| 0 | Reference |  |  |  |
| 1-3 | 0.54 (0.28-1.04) | 0.064 |  |  |
| ≥4 | 0.65 (0.34-1.26) | 0.202 |  |  |
| Radiation |  |  |  |  |
| No | Reference |  |  |  |
| Yes | 1.08 (0.63-1.87) | 0.772 |  |  |
| Tumor size | |  |  |  |
| <5cm | Reference |  | Reference |  |
| ≥5cm | 1.98 (1.03-3.82) | 0.04 | 1.98 (1.03-3.82) | 0.04 |
| Unknown | 1.73 (0.96-3.11) | 0.069 | 1.73 (0.96-3.11) | 0.069 |
